# Fecal microbiota in the female prairie vole (*Microtus ochrogaster*)

**DOI:** 10.1371/journal.pone.0190648

**Published:** 2018-03-26

**Authors:** J. Thomas Curtis, Senait Assefa, Amie Francis, Gerwald A. Köhler

**Affiliations:** 1 Department of Pharmacology and Physiology, Oklahoma State University Center for Health Sciences, Tulsa, Oklahoma, United States of America; 2 Department of Biochemistry and Microbiology, Oklahoma State University Center for Health Sciences, Tulsa, Oklahoma, United States of America; Oregon Health and Science University, UNITED STATES

## Abstract

We examined the fecal microbiota of female prairie voles. This species is socially and, likely, sexually monogamous, and thus serves as a valuable model in which to examine the interaction between the microbiota-gut-brain axis and social behavior. At present, little is known about the gastrointestinal microbiota of prairie voles; therefore, we performed a first characterization of the fecal microbiota using 16S rRNA gene amplicon sequencing. Semiconductor sequencing technology on an Ion Torrent PGM platform was used to assess the composition of fecal microbiotas from twelve female prairie voles. Following quality filtering, 1,017,756 sequencing reads were classified from phylum to genus level. At the phylum level, Firmicutes, Bacteroidetes, and Saccharibacteria were the predominant taxa, while the Bacteriodales, Erysipelotrichaceae, Ruminococcaceae, and Lachnospiraceae contributed the most dominant microbial groups and genera. Microbial community membership was most similar between vole sibling pairs, but consideration of taxon abundances weakened these associations. The interdependence of host factors such as genetics and behavior with the gastrointestinal microbiota is likely to be particularly pronounced in prairie voles. Our pilot characterization of the prairie vole intestinal microbiota revealed a microbial community composition remarkably consistent with the monogastric alimentary system of these rodents and their diet rich in complex plant carbohydrates. The highly social nature of these animals poses specific challenges to microbiome analyses that nonetheless are valuable for advancing research on the microbiota-gut-brain-behavior axis. Our study provides an important basis for future microbiome research in this emerging model organism for studying social behavior.

## Introduction

Commensal relationships, including those between a host and its associated microbiota, are ubiquitous in the animal kingdom. Complex microbiotas have been found in a variety of taxa including nematodes [1] and insects [2], and likely are present in all vertebrates (cf. [3]). In humans, the total number of microbial cells on and in a given individual is estimated to be two to three times greater than that of the person’s own human cells. In fact, the combined mass of the human microbiota is equivalent to that of the brain [4, 5]. By far the largest component of the human microbiota, the intestinal microbiota could be considered the equivalent of another “organ system” (e.g., [6, 7]). In this sense, the term “microbiota-gut-brain axis” has been coined to reflect the involvement of intestinal microbiota in regulating behavior [8, 9].

The role of the intestinal microbiota in both normal functioning and in the development of a variety of pathologies has been studied extensively in rats and mice. A PubMed search using microbiota and either rat or mouse returned over 4,300 hits. This is not surprising since these two species are most commonly used in animal research. However, for some lines of behavioral research rats and mice may not be the best models. Over the past thirty years, Microtine rodents, and in particular, monogamous prairie voles (*Microtus ochrogaster*) have become arguably the premier animal in which to study social bonding, especially social bonding between mates [10, 11]. Sexually naïve prairie voles of both sexes are highly social and appear to avoid isolation. These animals actively seek contact with conspecifics of either sex, but after successful mating both sexes develop strong aversive responses toward strangers, preferring instead to remain in close contact with their mate [12, 13]. The central processes that underlie this behavioral transition are well-studied and include brain regions associated with reward processing and aggression, and a variety of neurotransmitter and neuromodulator systems including dopamine, oxytocin, vasopressin, γ-amino butyric acid (GABA), and opioids [14–19]. More recently, it has been shown that pair-bonding between adult prairie voles involves epigenetic changes in some of these systems [20].

Growing evidence suggests that processes mediated by the intestinal microbiota can significantly influence behavior via actions within the central nervous system [21, 22]. Microbes and their metabolites have at least two routes by which they can influence behavior: direct communication with the brain via vagus nerve afferents [23], and indirectly via metabolic and immune factors delivered to the brain by the circulatory system [22, 24]. Microbiota-mediated influences on the central nervous system appear to help regulate normal functions such as development of the immune system and control of feeding [25, 26], and may contribute to a number of psychopathologies including impaired cognition, depression, autism, mood and eating disorders, and even posttraumatic stress disorder (PTSD) [21, 27, 28].

Given the rapidly growing list of behaviors that are affected by the microbiota, it is reasonable to expect that vole social behavior could be affected by the intestinal microbiota as well. There are several routes by which such effects might arise. First, the microbiota is a major source of neuroactive molecules and neurotransmitter precursors and thus could affect peripheral input to the brain and neural metabolism [28, 29]. Second, it has been shown that changes in the microbiota can alter the expression of some types of neurotransmitter receptors [23], including receptors involved in pair-bonding [19]. And finally, there is the possibility that microbial products may affect epigenetic changes [30] such as those involved in pair-bonding [20]. For example, among the more prominent microbial products are short-chain fatty acids, including butyrate which inhibits histone deacetylase that mediates epigenetic changes associated with pair-bonding [20]. Thus, changes in the microbiota could alter vole social behavior by altering neurotransmitter production, receptor expression and/or the epigenetic changes that accompany vole pair-bonding.

Examining a potential role for intestinal microbiota in vole social behavior will require at least a basic understanding of its composition. In contrast to the number of studies involving intestinal microbiota in rats and mice, there are relatively few for voles ((bank voles (*Myodes* = *Clethrionomys glareolus*) [31]; (*Microtus montebelli* and *M*. *arvalis*) [32]), and prairie voles [33]). To help fill this gap, here we describe the core microbiota of fecal samples from female prairie voles, including comparisons of the microbiotas from animals with different parents. These results should aid in the design and interpretation of experiments assessing a role for microbiota in social bonding.

## Materials and methods

### Vole husbandry

A laboratory breeding-colony descended from a southern Illinois population is housed in USDA approved facilities with routine care provided by Animal Resources personnel and is monitored daily. Breeding pairs are housed in plastic cages containing pine chip bedding with timothy hay as nesting material. *Ad libitum* food (Purina rabbit chow supplemented with sunflower seeds) and water are available. Animals are maintained at 21°C with a 14:10 light:dark cycle. Prairie voles are stressed by isolation [34], so after weaning at 20–21 days of age, pups in this study were housed in same-sex sibling pairs. Weaned males are kept in a separate room from females and breeder pairs. Veterinary staff is available for consultation regarding animal health and welfare. All experimental manipulations and animal handling procedures were approved by the Oklahoma State University Center for Health Sciences Institutional Animal Care and Use Committee.

### Subjects and sample collection

Subjects were 12 sexually-naïve adult (>60 days of age) female prairie voles of the F2 or F3 generations relative to the most recent out-crossing with wild stock. For sample collection, each female was weighed and then was placed alone in a clean cage containing a small amount of bedding, a few food pellets, and a drinking water source. One to three hours later, 4–6 fecal pellets were collected from each cage. Forceps used to pick up fecal pellets were rinsed in 100% ethanol between animals to minimize cross-contamination. The fecal pellets from each female were pooled and stored at -80° until used for assays. Only females were used as this study was a prelude to a study examining the effects of cyclic changes in estrogen on intestinal microbiotas.

### Fecal sample processing

DNA was extracted from approximately 40–90 mg of prairie vole fecal samples using the ZR Fecal DNA MiniPrep Kit (Zymo Research, Irvine, CA). A Mini-Beadbeater-96 (Biospec Products, Bartlesville, OK) was employed for bacterial cell disruption during the DNA isolation procedure. Following two bead beating cycles of 2 min duration at 2,400 oscillations/min, the resulting fecal homogenates were processed for fecal genomic DNA isolation according to the manufacturer’s instructions.

The concentrations of the isolated DNAs were determined using a Qubit 2 Fluorometer (Life Technologies, Thermo Fisher Scientific) in conjunction with the Qubit dsDNA HS Assay Kit (Thermo Fisher Scientific). The DNA extracts also were evaluated for quality by agarose (1%) gel electrophoresis in 1× Tris-Acetate-EDTA buffer. Samples that showed extensive smearing because of low molecular weight DNA fragments were discarded and those DNA isolations were repeated. Isolated DNA samples with bands between 8 kb to larger than the 10 kb band of the TriDye 2-log DNA Ladder (New England Biolabs) were deemed good quality and stored at -20°C before being used as templates for next-generation sequencing library preparation.

### PCR amplification and sequencing library preparation

An Ion 16S Metagenomics Kit (Life Technologies, Carlsbad, CA) was used to amplify 16S amplicons from the isolated fecal DNAs. This kit employs two primer sets targeting the V2-4-8 and V3-6, 7–9 hypervariable regions. Following manufacturer’s instructions, PCR amplifications were prepared with 1–3 μl of the extracted fecal DNAs as templates for two separate 30 μl reactions using the aforementioned primer sets and run on a PTC 200 DNA Engine thermocyler (BioRad, Hercules, CA). PCR conditions for construction of all sequencing libraries consisted of one initial cycle at 95°C for 10 min; 25 cycles of 95°C for 30 s, 58°C for 30 s, and 72°C for 20 s; and a final incubation at 72°C for 7 min. Following amplification, the presence of PCR products from the two separate reactions was confirmed by running aliquots of the reactions on 2% agarose E-gel double comp gels (Life Technologies). Strong amplicon bands at the expected size ranges were detected in all samples, indicating that reaction inhibition was absent or minimal. No-template-control reactions did not yield amplification products. Pooled PCR products from each sample were purified using AMPure XP magnetic beads (Agencourt, Beckman Coulter, Inc., Indianapolis, IN), eluted in 15μl of nuclease free water and quantitated using a Qubit dsDNA HS kit (Life Technologies). The sample amplicons were end-repaired using the Ion Plus Fragment Library kit following the manufacturer’s instructions. Repaired amplicon products were purified using AMPure XP magnetic beads and eluted in 25μl low Tris-EDTA (low-TE) buffer. Subsequently, these repaired and purified amplicons were barcoded according to sample assignments using the Ion Plus Fragment Library kit in conjunction with Ion Xpress Barcode Adaptors 1–16 kit (Life Technologies). Following another AMPure clean-up and elution step (20 μl), each DNA library was quantified by quantitative PCR (qPCR) using the Universal Library Quantitation Kit (Life Technologies). Each quantified library was diluted to a final concentration of 10 pM. Equal volumes of each dilution then were pooled for template preparation and enrichment on the One-Touch 2 and One-Touch ES systems (Life Technologies), respectively. The Ion PGM Template OT2 400 kit was used for preparing template-positive Ion PGM OT2 400 Ion Sphere Particles (Life Technologies) on these systems. Finally, next-generation sequencing of the 16S rRNA gene fragment libraries was performed on an Ion PGM System (Life Technologies) using Ion PGM 400 sequencing reagents and Ion 318v2 chips following the manufacturer’s instructions.

### Next-generation sequencing data analysis and visualization

Following sequencing, raw sequence data for each sample were downloaded from the Ion Torrent server as unaligned/unmapped, demultiplexed reads (fastq format, barcodes and adapters removed) and imported into CLC Genomics Workbench (Qiagen, Redwood City, CA) for length (175 to 300 nt) and quality trimming using default parameters for Ion Torrent data (quality limit 0.05; maximal two sequence ambiguities). Further processing of the resulting 1,039,149 sequences was adapted from an analysis pipeline recently developed by Barb and coworkers for Ion 16S Metagenomics Kit sequencing data [35]. Adaptations were necessary to accommodate differences in sample types (mock vs. fecal microbiota) and bioinformatics equipment and software—details on sequence processing and data analysis are available in S1 Table. Briefly, the twelve individual sample fasta files received sample-specific read labels and were combined into one file using QIIME version 1.9.1 [36]. The combined sequences were aligned to the SILVA 16S rRNA database (Release 128; [37]) in mothur [38]. By passing the flip option in the align.seqs command, this process enabled us to separate forward and reverse reads from each other. Following an additional quality control step (minimum alignment length 175 nt), summary.seqs reports generated from forward and reverse reads were imported into Microsoft Excel spreadsheets and reads were parsed and counted according to their alignment coordinates using Excel pivot tables. SILVA alignment coordinates were translated to *Escherichia coli* 16S rRNA gene coordinates [39] using an Excel spreadsheet query (see S2 Table for coordinate translations and S3 Table for binning parameters.) in order to map reads to their respective hypervariable regions (V2, V3, V4, V6-7, V8, and V9). Reads that failed to map to any of these regions were discarded. Read frequency mapping plots for forward and reverse reads were generated using the ggplot2 system for R (see S1 Fig). Read-hypervariable region assignments in Excel were used to generate specific accnos files for use with the get.seqs command in mothur in order to demultiplex the combined sequences fasta file into hypervariable region-specific forward and reverse read fasta files. Since the primer sequences used in the Ion 16S Metagenomics kit are not published, we trimmed the first 20 bases from the 5’ end of each read using CLC Genomics Workbench, emulating the approach by Barb et al. [35]. Trimmed sequence fasta files were imported into QIIME and sequence chimeras were removed using the usearch61 method, *de novo* and in combination with the SILVA version 128 16S rRNA reference database. Following pilot analyses with twelve quality-controlled fasta files, we decided to combine the forward and reverse reads of each of the six 16S sequence regions to reduce the complexity of subsequent analysis steps. The S3 and S5 Tables contain detailed information on read numbers per hypervariable region or sample.

The resulting six sets of sequence reads were classified individually in QIIME version 1.9.1 [36, 40] by open reference operational taxonomic unit (OTU) picking using the usearch61 method in conjunction with the SILVA 16S rRNA database (Release 128; 97% identity level) and RDP Classifier for taxonomic assignments at 90% cutoff [37, 39, 41, 42]. The open-reference OTU picking strategy was chosen over closed-reference picking in order to be able to detect novel diversity in the vole microbiota, despite the drawback that open-reference picking precluded simultaneous analysis of multiple hypervariable regions. The resulting OTU tables derived from the six hypervariable region read sets were filtered to remove spurious OTUs (number of sequences < 0.005%). S6 Table encompasses OTU tables for each hypervariable region. A core set of diversity analyses was run in QIIME on each hypervariable region read set to compute the phylotype diversities (alpha, beta diversities) of the fecal samples’ bacterial communities [36, 43, 44]. The even sampling (rarefaction) depth of each of the six reads sets was adjusted to the lowest number of reads in the samples (for details see S5 Table.). For each read set, average linkage clustering (UPGMA) trees were constructed with jackknife support from weighted and unweighted UniFrac distances to cluster samples. For depiction, the resulting trees were formatted and labeled in CLC Genomics Workbench. A combined hypervariable region V2, V3, V4, V6-7, V8, and V9 OTU table for each sample was generated by summing the OTUs derived from the six sequenced regions. This OTU table was filtered to a minimum count fraction of at least 0.0005 (≥0.05% relative abundance) and then imported into MEGAN6 to prepare phylogenetic trees [45]. A circular cladogram visualizing OTUs and their relative abundances at up to six taxonomic levels was constructed in GraPhlAn [46]. For additional diversity analyses of the overall fecal microbiota taxonomic profiles of individual and grouped animals (e.g., sibling pairs), a summed OTU table was generated combining the even sampling OTU tables of the six hypervariable regions (see S6 Table). QIIME non-phylogenetic core/jackknifed diversity analyses and Statistical Analysis of Metagenomic Profiles (STAMP) [47] software were used to analyze the combined taxonomic profiles.

### Statistical analyses

Standard parametric statistical analyses (Excel, Microsoft) were used to examine vole demographics. F-tests were used to ensure homogeneity of variance, after which unpaired t-tests were used to assess potential age and mass differences between females from the two generations. Demographics data are presented as mean ± se.

Intestinal microbiota profiles of the prairie voles were analyzed in STAMP with ANOVA and Games-Howell post-hoc tests. For multiple test corrections, the Bonferroni procedure was used. The effect size threshold was ≥ 0.80. Statistical analysis results with p < 0.05 were considered significant. Sibling pair distances were compared using two-tailed Student’s t-tests with calculation of non-parametric p values using 1000 Monte Carlo permutations and Bonferroni correction.

## Results

The composition of fecal microbiotas from 12 female prairie voles was assessed via 16S rRNA gene sequencing using the commercially available 16S Metagenomics Kit tailored for the Ion Torrent PGM system. This technology was successfully employed and evaluated in previous studies. For example, Zeber-Lubecka at al. studied the effect of a probiotic and the mode of delivery on the gut microbiota in preterm infants [48] and Sperling et al. surveyed tick microbiomes using the 16S Metagenomics kit [49]. Barb and coworkers used the kit to sequence 16S rRNA mock samples and developed an analysis pipeline for the multiple hypervariable regions (V2, V3, V4, V6-7, V8 and V9) targeted by the proprietary primer pairs utilized during sequencing library preparation [35]. Since this analytical approach provided flexibility in various aspects of 16S rRNA-based microbiota analysis (e.g., choice of 16S rRNA reference database for taxonomic assignment), we adapted the workflow to our hard- and software infrastructure. When compared to single hypervariable region-based approaches, the interrogation of multiple hypervariable regions poses unique challenges, but might also deliver a deeper assessment of microbial diversity.

### Animal demographics

The average age on the day of sample collection of the females used in this study was 83.2 ± 3.3 days (range 66–99) and the average mass was 35.3 ± 0.9 g (range 30.4–40.0g). Each of the six pairs of female siblings was derived from a different pair of breeders. Three sibling pairs were from the F2 generation and 3 pairs were from the F3 generation relative to the most recent out-crossing of the breeding colony. There were no age (t_10_ = 0.14, p = 0.89) or mass (t_10_ = 0.16, p = 0.88) differences between the females from the two generations.

### Analysis of multiple 16S rRNA hypervariable regions

Library preparation with the 16S Metagenomics kit yields bidirectional sequencing templates from two primers sets (V2-V4-V8 and V3-6,7–9) targeting the hypervariable regions V2, V3, V4, V6-7, V8, and V9 that are sequenced unidirectional, thus forward and reverse reads from the six targeted regions will be generated simultaneously from each sample. Sequencing runs can be multiplexed by using a different barcoded adapter (Ion Xpress^™^ Barcode Adapters) for each sample during adapter ligation.

Since the actual 16S primer sequences are proprietary, we adapted the approach by Barb et al. [35] and separated forward and reverse reads after alignment to the SILVA 16S rRNA gene reference alignment (version 128) using mothur [37–39]. Subsequently, the reads were mapped to respective hypervariable regions using the *E*. *coli* 16S rRNA gene as positional template as described in Material and Methods (see also S1 to S4 Tables and S1 Fig). While this procedure was relatively straightforward, with a very low number of unmapped reads, it revealed substantial differences in the relative representation of hypervariable regions. Further quality control included trimming of 20 nucleotides at the 5’ ends of all reads to reduce potential primer biases during subsequent analyses and removal of sequence chimeras, resulting in a total of 1,017,756 reads. Individual sample and average read percentages for the targeted hypervariable regions are summarized in the supporting information (S3 and S4 Tables). Open-reference OTU picking in QIIME was used on the combined forwards and reverse read sets of each hypervariable region, resulting in a total number of 837,386 reads with taxonomic assignment. The highest average number of OTU-assigned reads originated from hypervariable regions V3 (mean 34.3%) and V8 (32.7%) while V2 (2.8%) and V9 (2.9%) showed the lowest percentages. V4 and V6-7 yielded 8.3% and 19.0%, respectively. S2 Fig and S5 Table show the distribution of reads with taxonomic assignment for each sample.

Our analysis pipeline included several quality control steps such as read length, Phred score, reference alignment, hypervariable region binning, chimera check, and OTU picking with taxonomic assignment that led to substantial reduction in read counts per sample (up to 25% after the initial length and quality trimming). The contributions to overall read counts varied widely between the different hypervariable regions, albeit the between sample distributions were remarkably similar (see S2 Fig).

### Predominant microbial taxa

**Fig 1:**
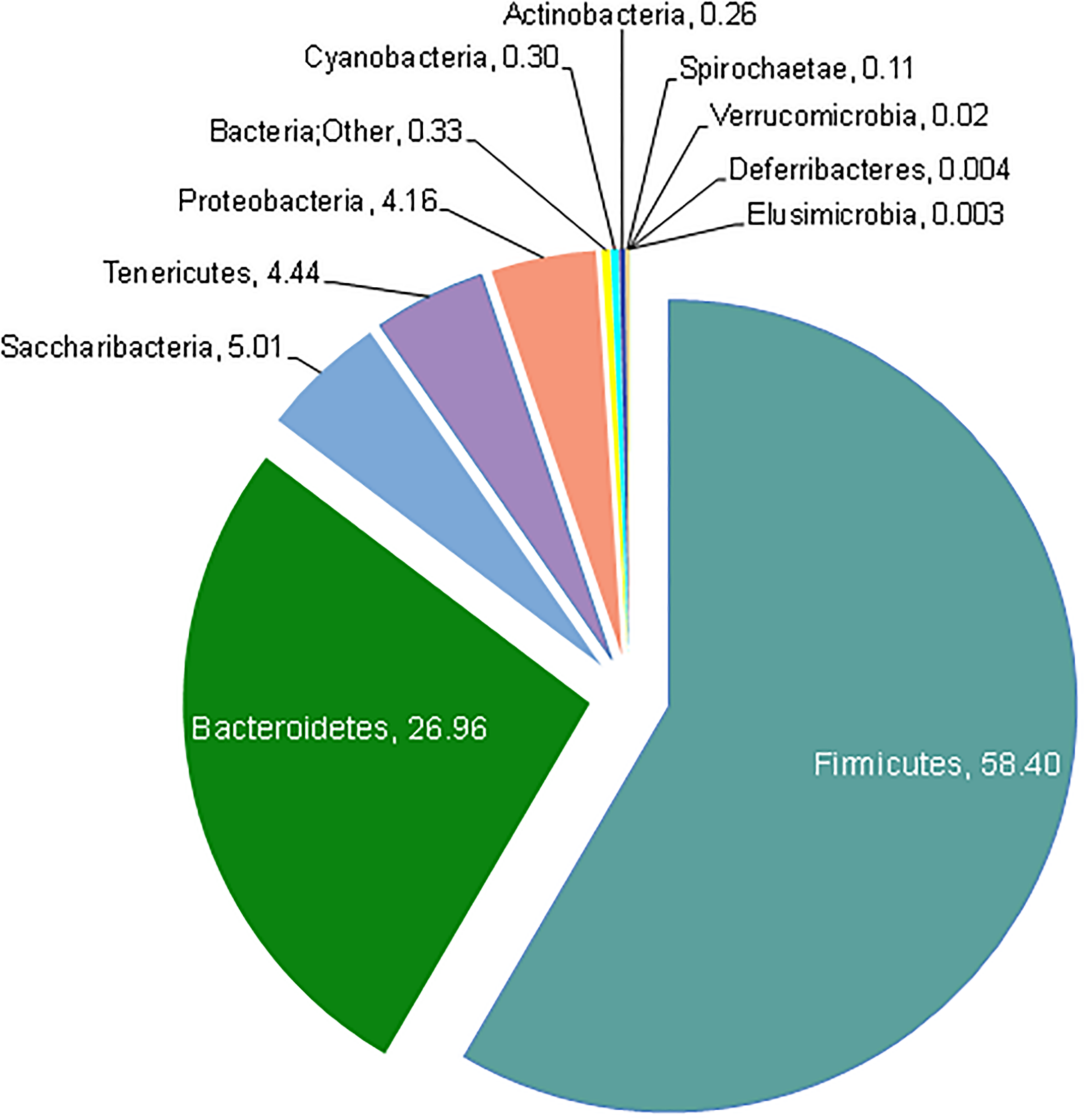
Average abundance of bacterial phyla in the prairie vole intestinal microbiota. The pie chart shows the average percentages of the eleven most abundant phyla in fecal samples from twelve female voles. The Firmicutes is clearly the most dominant phylum, the second- and third-most frequent phyla are the Bacteroidetes and the candidate phylum Saccharibacteria (formerly known as TM7), while the remaining phyla comprise less than 10%. Only about 0.33% of reads did not receive a phylum assignment (Bacteria; Other) using the OTU picking approach described in the text. Numbers indicate the relative abundances in percent of the classified OTUs at phylum level.

Open-reference OTU picking revealed eleven phyla in fecal pellets from all of the animals tested (see Fig 1). The Firmicutes were the predominant phylum (~58%). Bacteriodetes and Saccharibacteria were the second and third most abundant (27% and 5% respectively). The remaining eight phyla (Tenericutes, Proteobacteria, Cyanobacteria, Actinobacteria, Spirochaetae, Verrucomicrobia, Deferribacteres, and Elusimicrobia) combined comprised < 10% of the total.

**Fig 2:**
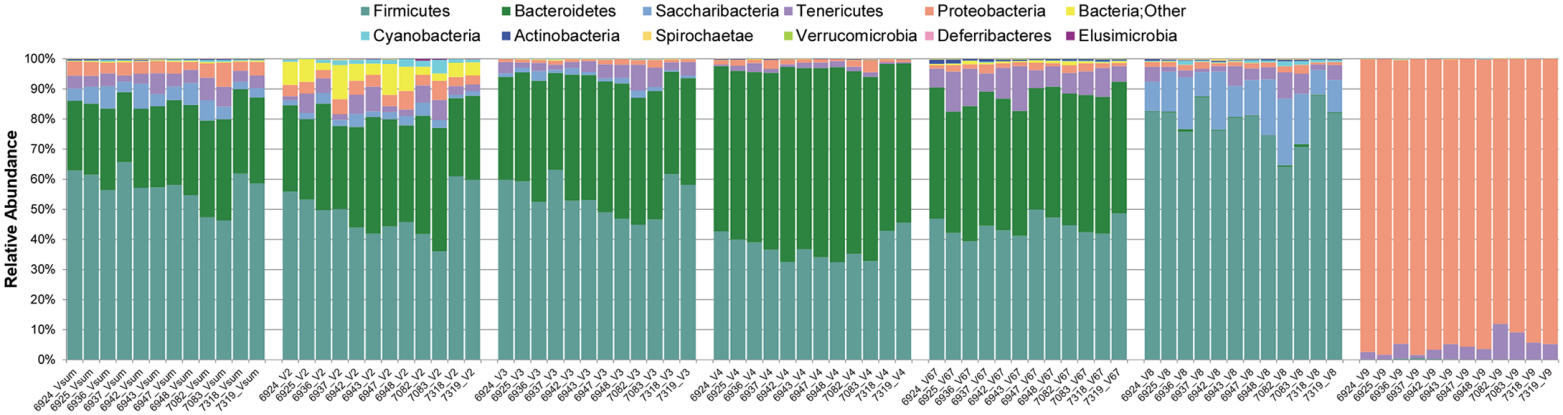
Stacked bar graphs showing the relative abundance of phyla by hypervariable region. The left-most panel shows the summed-up phyla representation of all sequenced hypervariable regions (Vsum) for the twelve samples, while the following panels depict the relative abundances of the individual regions V2, V3, V4, V6-7, V8, and V9, respectively. Differences in the representation of individual phyla are prominent, especially in the top five phyla, Firmicutes, Bacteroidetes, Saccharibacteria, Tenericutes, and Proteobacteria. Reads without phylum assignments (yellow bar segments; Bacteria;Other) appeared predominantly in V2.

Some reads (overall average 0.33%) were not assigned to a phylum using the methods and parameters used during OTU picking (see S1 Table). Interestingly, the vast majority of these reads originated in the V2 data set with an averaged proportion of 5.25% (see also Fig 2), while the other hypervariable region data sets each harbored <0.7% reads without phylum assignment. Phylum representation differed quite strongly between hypervariable regions (Fig 2). V9 almost exclusively showed Proteobacteria and to lesser extent Tenericutes. A large number of reads assigned to Saccharibacteria appeared in the V8 read set, while this taxon was completely absent in the OTUs associated with V4, V6-7, and V9. The most dominant phylum Firmicutes was strongly represented in V2, V3, V4, V6-7, and V8. This profile was shared by the Bacteroidetes, the second-most abundant phylum, with the exception of the V8 read set, which had very few reads assigned to this taxon. Bacteroidetes surpassed the Firmicutes in V4 in all samples (Fig 2)

**Fig 3:**
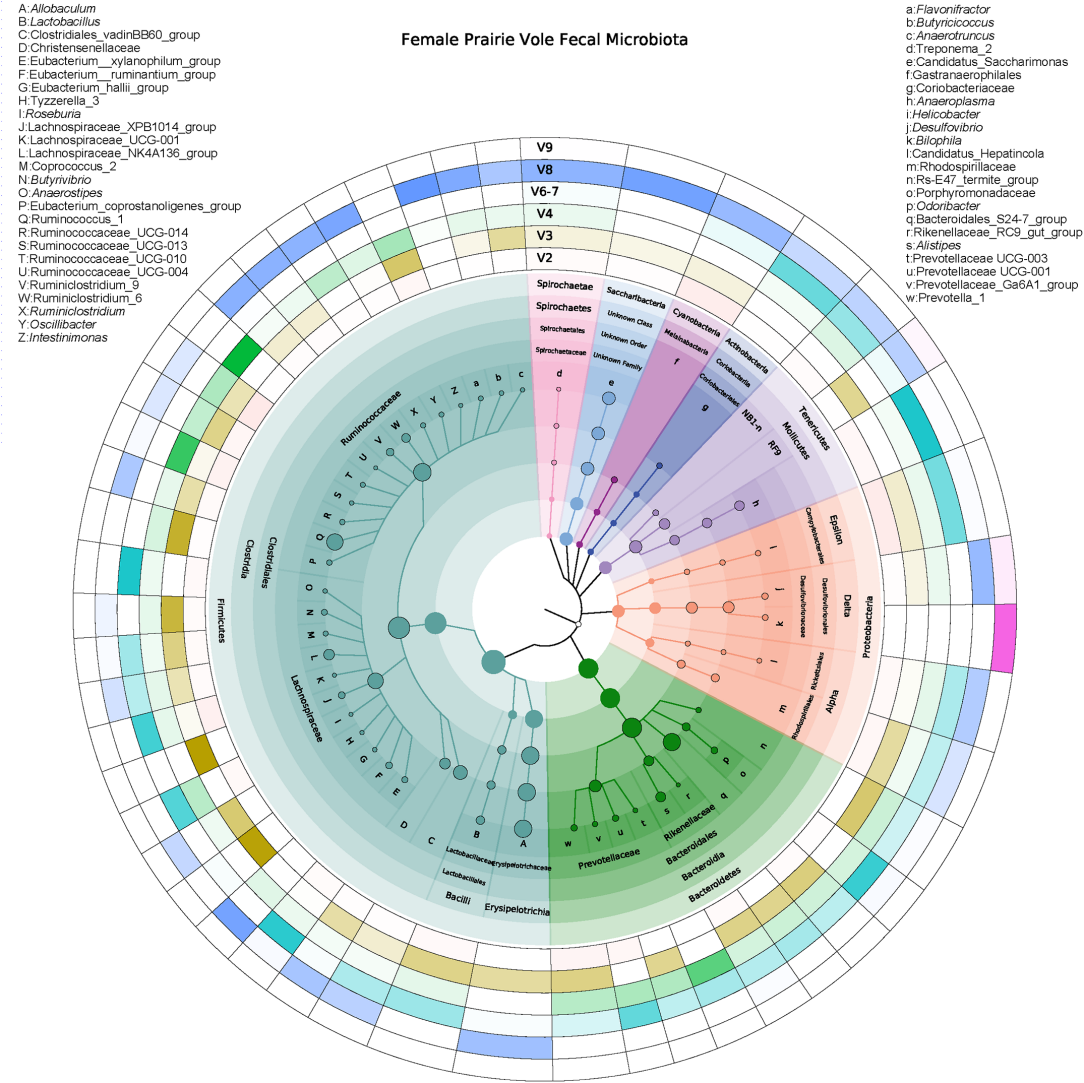
Circular cladogram of 16S rRNA gene phylotypes in female prairie vole fecal microbiota. Bacterial phylotypes (all with relative abundances of >0.05%) are depicted with clade-specific background and node coloring from phylum to genus level or the lowest available level with a distinct taxonomic assignment in the SILVA 16S database (release 128). Beginning at the phylum level, node sizes correlate with the square root of the average relative abundances of the individual phylotypes in the samples from all animals. The six outer rings show heatmaps indicating the relative abundances of taxon-specific reads within the hypervariable regions V2, V3, V4, V6-7, V8, and V9. Comparison of color intensities in the ring segments reveals how some phylotypes were detected in several or only in very few hypervariable region data sets. For example, *Bilophila* (k) appeared only in V9, while the majority of phyla were present in multiple hypervariable region OTU tables.

At the family and genus levels, the Firmicutes were the most diverse phylum, and included 27 assignments at the genus level in the cladogram depicted in Fig 3. This cladogram is restricted to OTUs with a relative abundance of 0.05% and above. The next most diverse phylum was the Bacteriodetes, which included five families and seven genus level assignments (Fig 3). Most Bacteroidetes were classified as members of the Bacteroidales S24-7 group, followed by the genus *Alistipes*. Among the OTUs with ≥ 0.05% abundance, Proteobacteria were represented as Alpha-, Delta-, and Epsilon-proteobacteria with the latter showing Helicobacter as the dominant genus. In the Alpha-proteobacteria clade, Candidatus Hepatincola was the only genus, while the Delta-proteobacteria included the two genera *Bilophila* and *Desulfovibrio*. Among the remaining phyla with at least 0.05% relative abundance, the Tenericutes encompassed the genus *Anaeroplasma* and the two order-level groups NB1-n and RF9. The Actinobacteria and Spirochaetae included the single family Coriobacteriaceae and genus-level Treponema group 2, respectively. Interestingly, as in other intestinal microbiota studies, the cyanobacteria detected in this study do not appear to represent allochthonous photosynthetic “contaminants”, but rather are members of the Gastranerophilales, a recently described order of the cyanobacterial sibling phylum/class Melainabacteria [50, 51], As the third-most abundant phylum, the Saccharibacteria (TM7) were classified to genus_level Candidatus Saccharimonas.

### Diversity analyses of vole fecal microbiota

Phylogenetic and non-phylogenetic alpha-diversity analyses were conducted using the OTU tables derived from the six 16S rRNA hypervariable regions. Sample read sets were rarefied to the lowest read count in each data set (see S3 Table). S3 Fig shows phylogenetic diversity (PD_whole_tree) as well as Shannon index and observed OTUs rarefaction curves for all samples and hypervariable regions. These curves indicate adequate sequencing depth in read-rich hypervariable regions (e.g., V3, V8). The lower counts in the V2 read set and especially in the V9 set reduced confidence in alpha diversity measures because rarefaction curves were the furthest from reaching a plateau. Comparison of alpha-diversity measures did not reveal significant differences between of sibling pairs. When alpha diversities of the filial generations F2 and F3 were compared only two analyses revealed p values <0.05: number of OTUs in the V3 data set (p = 0.043) and phylogenetic distance in the V8 data set (p = 0.035).

**Fig 4:**
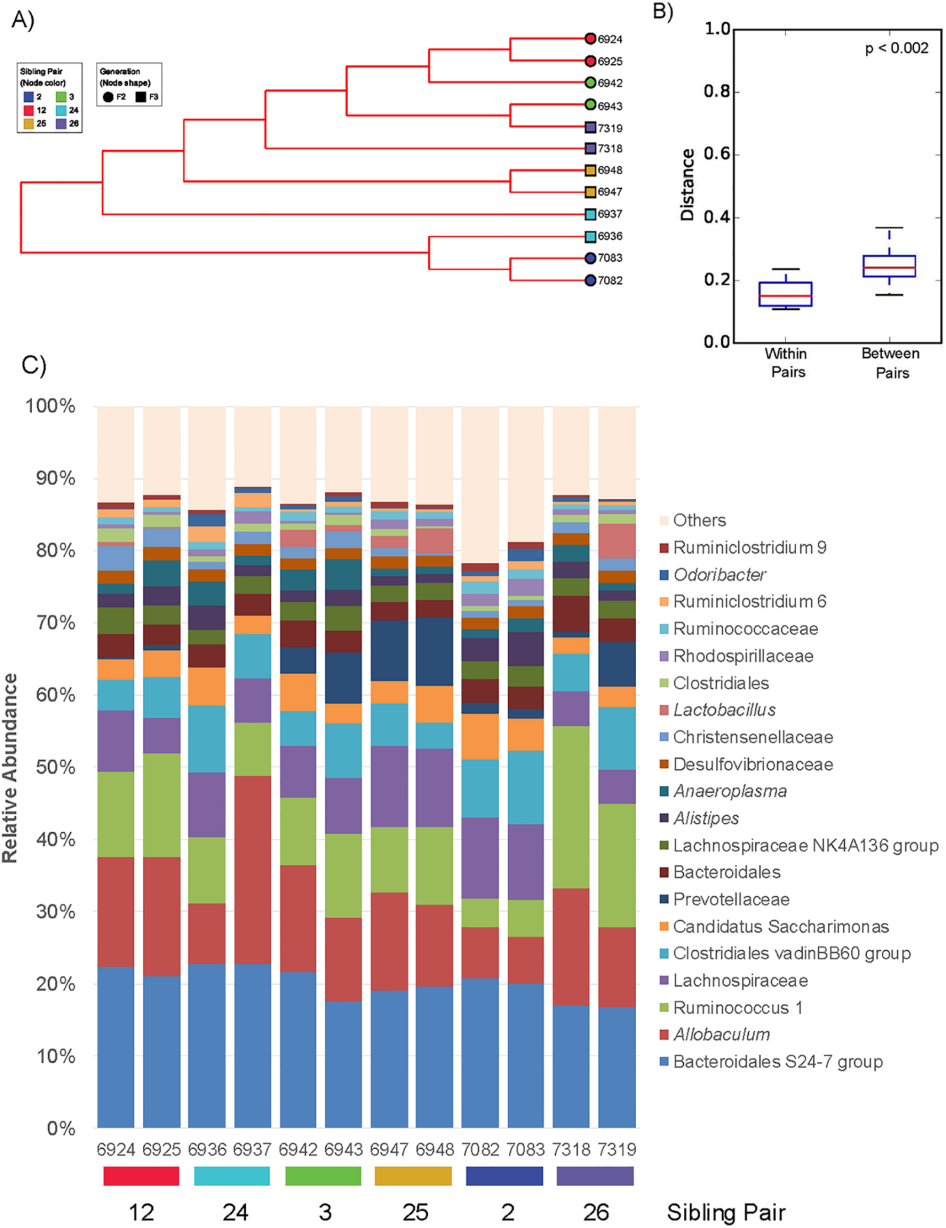
Non-phylogenetic beta-diversity of vole fecal samples. The between sample similarities of vole fecal microbiotas at the genus level were calculated by beta diversity analysis using the Bray-Curtis dissimilarity metric [52] which takes OTU abundance into account. UPGMA clustering of the samples is shown in a cladogram with jackknife support (A). Distance comparisons within and between sibling pairs indicated that the overall composition of microbiotas in fecal pellets from female prairie voles at the genus level was more similar between siblings than between sibling pairs (B). In the cladogram, each label color represents a different sibling pair, while the shapes indicate F2 or F3 generations relative to the most recent out-crossing of the breeding colony with wild stock. While some animals clustered very close with their sibling (pairs (12, 25, and 26), other siblings were split and clustered with unrelated animals. The differential abundances of bacterial groups in the individual microbiotas likely caused this non-sibling clustering, as the comparison of unweighted versus weighted UniFrac analyses also suggests (see S4 Fig). The stacked bar graph on the 20 most abundant genus-level OTUs illustrates how differential abundances of bacterial groups within sibling pairs could lead to association with non-sibling animals. For example, the clustering of animal 6936 with animals 7082 and 7083 could be driven by the relatively low abundance of *Allobaculum* versus high levels of Clostridiales vadinBB60 and Candidatus Saccharimonas (C) in these animals. Statistical analysis: non-parametric t-test with 1000 Monte Carlo permutations and Bonferroni correction, significant p value < 0.05. The Jackknife support values were all above 75%.

Beta-diversity analyses revealed that, in general, the fecal microbiotas were more similar between siblings than between unrelated sibling pairs. Non-phylogenetic UPGMA clustering and distance comparisons using the Bray-Curtis metric were performed on the genus-level OTU table resulting from read summation of all analyzed variable regions (see Fig 4A and 4B). Phylogenetic distance analyses using weighted and unweighted UniFrac measures were only possible for the individual variable regions because of the limitations imposed by *de novo* clustering during open reference picking. UniFrac-based UPGMA clustering cladograms and distance comparisons between sibling pairs are shown in S4A and S4B Fig. Comparison of filial generations yielded no significant differences in the distances among and between F2 and F3 generations. Sibling pair comparison plots on the other hand revealed that the within pair distances were significantly lower than the between sibling pair distances in the genus level Bray-Curtis distance matrix (see Fig 4B) and all variable region unweighted UniFrac distance matrices (S5A Fig). This also held true for the weighted UniFrac distance comparisons, with the exception of the plots for V8 and V9 which showed no significant differences (S5B Fig). The unweighted UniFrac phylogenetic distance analysis, which does not take relative abundances of taxa into account, showed that the microbiotas of siblings accurately reflected sibling status in V2, V3, V4, V6-7 data, and to reduced extend in V8 data (S4A Fig). This analysis also showed that further groupings differed among variable regions. V9 clustering produced a low confidence cladogram that only partially revealed sibling pair clusters. Concerning distribution of animals by filial generation, no obvious pattern was found.

Overall, the beta diversity analyses using non-phylogenetic Bray-Curtis and the phylogenetic weighted/unweighted UniFrac metrics revealed that results are influenced not only by the metric employed, but also by the variable region from which the dataset originated. Phylogenetic analysis based on presence/absence of OTUs paired animals in concordance with biological relationships in all datasets, with the exception of V9. The abundance driven analyses (Bray-Curtis, weighted UniFrac), however, led to various rearrangements during clustering. Scrutiny of the microbiota compositions derived from the summed V2, V3, V4, V6-7, V8 and V9 dataset shown in Fig 4C illustrates how differential abundances between siblings may influence clustering.

At the genus level, the phylotype sequences of Bacteroidales S24-7 group (mean 20%, min 17%, max 23%), *Allobaculum* (mean 13%, min 7%, max 26%),and Ruminococcus 1 (mean 11%, min 4%, max 23%) represented the most dominant annotations overall (see also Fig 4C). The Bacteroidales S24-7 group were the leading group in nine animals. In the sibling animals 7318 and 7319 Ruminococcus 1 ranked the highest (22% and 17%, respectively) while *Allobaculum* reads outnumbered the aforementioned groups in one animal (26% in 6937). Fig 4C provides an overview of the top 20 genus-level taxa in the female prairie vole fecal microbiota.

**Fig 5:**
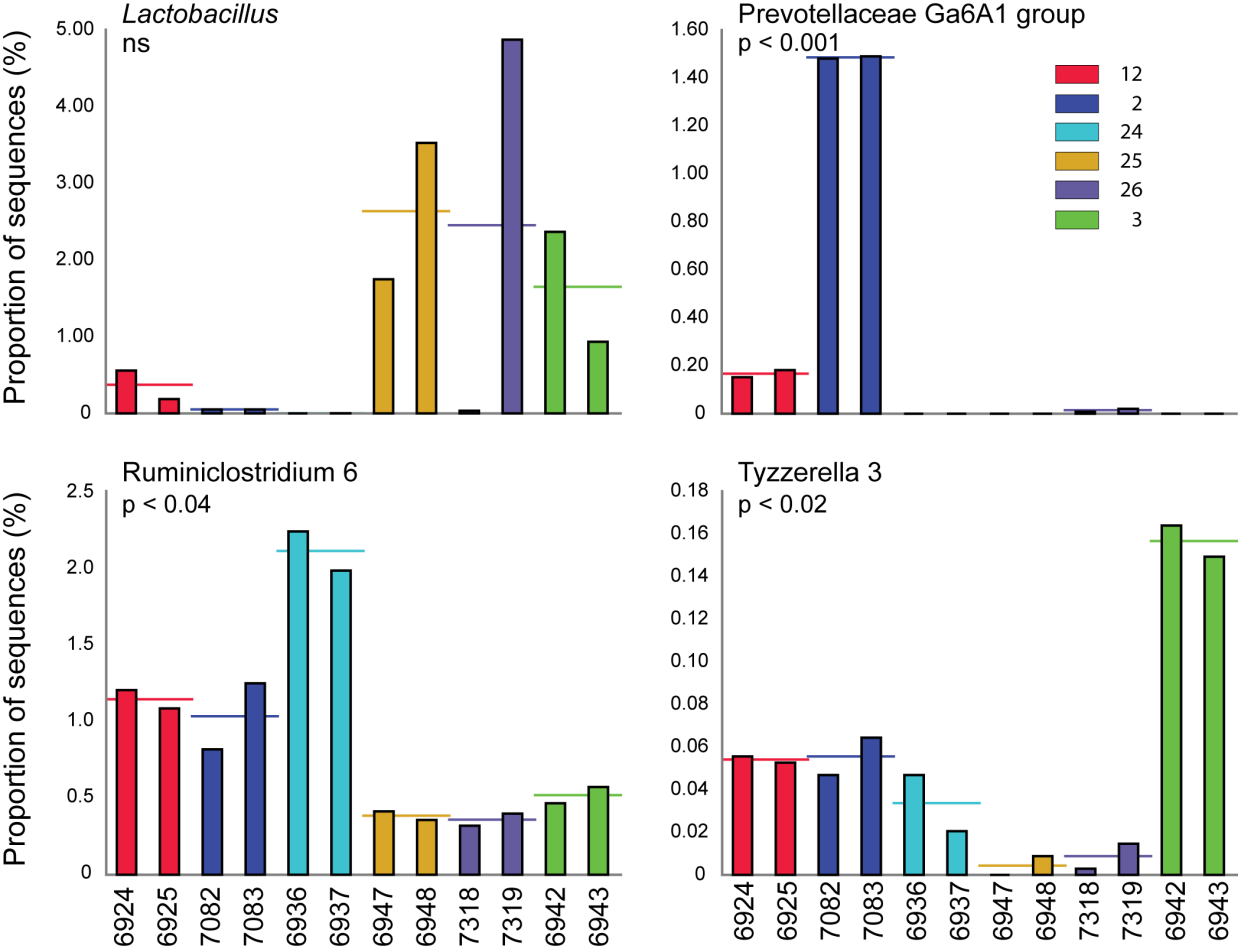
Comparative abundances of *Lactobacillus*, Prevotellaceae, *Ruminiclostridium*, and *Tyzzerella* phylotypes. Bars show the relative abundances of the genus level phylotypes *Lactobacillus*, Prevotellaceae Ga6A1 group, Ruminiclostridium 6, and Tyzzerella 3. Large variations in the fecal lactobacilli are seen between animals. Differences in Prevotellaceae Ga6A1 group, Ruminiclostridium 6, and Tyzzerella 3 between sibling pairs were significant (ANOVA, Games-Howell post-hoc tests with Bonferroni multiple comparison correction; p values are indicated, ns: not significant).

Interestingly, the genus *Lactobacillus* varied strongly between animals, as indicated in Fig 5. In a recent study, we observed a similarly wide variation in *Lactobacillus* abundance in the gastrointestinal tracts of individual voles [33]: some animals had high levels of lactobacilli in the stomach and small intestine, which correlated with increased levels in the colon, in contrast to animals with consistently low levels in the upper and lower gastrointestinal tract.

Further analysis of microbiota profiles revealed significant differences in the sibling pairs in the proportion of sequences classified as Prevotellaceae Ga6A1 group, Ruminiclostridium 6, and Tyzzerella 3 (see Fig 5). This analysis also was restricted to sequences with at least one occurrence of 0.1% or higher abundance.

## Discussion

The present study focused on female prairie voles. Although similar microbiota characterization in males is planned, the current report is preliminary to examining the effects of female reproductive hormones on the intestinal microbiota. Unlike typical laboratory animals such as rats and mice, prairie voles do not display a spontaneous puberty. Rather, in the absence of chemosignals from a male, although fully grown, “adult” female voles essentially remain in diestrous with low but constant levels of circulating estrogen [53–56]. This allows us to characterize female microbiota while being confident that the animals all would have the same gonadal hormonal profile. Future studies will examine how the female intestinal microbiota might change after reproductive activation associated with exposure to males. To this end, our ultimate goal is to monitor microbiota changes over the time-course of reproductive activation. Thus, we focused on fecal microbiota as an index of status of the intestinal microbiota since fecal sampling reduces animal usage by providing for repeated measures in the same animals.

Semiconductor sequencing on the Ion Torrent PGM platform in combination with the 16S Metagenomics Kit was validated in at least two recent studies: Zeber-Lubecka and coworkers used and validated the approach in a study on the effects of probiotics and the mode of delivery on preterm infants [48] while Barb *et al*. developed an analysis pipeline for this multiple hypervariable region sequencing approach and validated it with bacterial mock communities [35]. The4 former study showed very low median bias in mock community sequencing [48]. The very detailed validation of the latter study revealed that the hypervariable regions V2, V3, V4, V6-7, V8, and V9 targeted with the 16S Metagenomics kit performed disparately in accurately representing the known mock samples—regions V3 and V9 showed the highest divergence from the mock sample compositions (at family and genus level), while V2 and V4 showed the lowest. We applied the same sequencing technology and adapted the data analysis pipeline of Barb et al. [35] to our bioinformatics infrastructure and sample type. For example, adjustments in read length filtering to 175–300 bases instead of 200–300 bases were indicated because of the presence of a high read abundance peak just below 200 bases in the prairie vole microbiota read length profile.

As in the Barb et al. study, comparison of OTUs across hypervariable regions clearly revealed compositional differences among the regions, with region V9 showing the lowest diversity. While we inspected and analyzed the results of individual hypervariable regions, we also combined the OTUs across all regions (e.g., at genus level) in an effort to build a consensus representation of the female prairie vole fecal microbiota. The latter approach is hampered by potentially double or multiple counting of individual 16S rRNA genes which could lead to artificial inflation of some taxonomic assignments. At present, we are not aware of a straightforward method to compute an unequivocal consensus across all regions. On the other hand, some phylotypes such as Saccharibacteria (present in the V2, V3, and V8 datasets at 1%, 10%, and 89%, respectively) and *Bilophila* (to 100% in V9) would have been missed in our study if we would have analyzed only a single region such as V4. In future studies, we will include mock communities as sequencing and data analysis controls; however, careful selection of community members will be necessary to avoid bias towards commonly used hypervariable regions such as V4 which could result in false negatives in OTUs exclusively covered, for example, by V9 amplicon sequencing. Mock communities that a tailored to or derived from a specific host such as the prairie vole probably will be the best choice in this regard.

Following the aforementioned analysis pipeline, taxonomic assignment of OTUs yielded this first survey on the microbial community structure in the prairie vole, an established model organism for social behavior research. Microbiotas in the mammalian intestine are strongly influenced by host diet and phylogeny [57]. In comparison to carnivory and omnivory, herbivory appears to be associated with increased bacterial diversity and preponderance of microorganisms capable of breaking down complex plant carbohydrates such as resistant starches and celluloses [57–59]. Anatomical characteristics such as high-crowned-rootless molars, an extremely large cecum, and a highly developed post-cecal spiral suggest that voles are primarily herbivorous [60]. A variety of field studies (summarized by [60]) suggest that prairie voles consume primarily the vegetative portions of plants which tend to be high in fiber but low in nutritional value. The predominant fecal OTUs identified in all prairie voles in our study are consistent with this assessment of the prairie vole dietary preferences. Members of the Ruminococcaceae and Lachnospiraceae are specialists in degrading complex plant material [61]. The genus *Ruminococcus* is one of the most dominant phylotypes in our study and several genera/groups (e.g. Lachnospiraceae NK4A136 and XBP1014 groups, *Butyrivibrio*) within the *Lachnospiraceae* appear to encompass a substantial portion of the vole microbiota. While no deeper classification of the *Ruminococcus* sequences was possible with the SILVA 16S database used in this study, a tentative association of the majority of these sequences with *R*. *flavefaciens* was detected using the Greengenes 16S rRNA database. *R*. *flavefaciens* is known to express a complex cellulolytic machinery (cellusome) and has been found as one of the main cellulolytic species in ruminant and non-ruminant herbivores [59, 62–64], including voles [32]. Interestingly, Ruminococcaceae and Lachnospiraceae were the most abundant families in the gut microbiota of wild mice, while these bacteria exhibited lower abundance in inbred mice [65, 66]. Furthermore, a recent metagenomic study on liver samples from field voles (*Microtus agrestis*), tundra voles (*Microtus oeconomus*), and bank voles (*Myodes glareolus*) also revealed large abundances in of the *Ruminococcaceae* and *Lachnospiraceae* [67]. In contrast to our study many clinically relevant genera were detected (e.g., *Bartonella*, *Francisella*), which is likely attributable to the fact that the liver samples were collected from wild animals.

Another predominant group of bacteria in the vole fecal microbiota, the *Allobaculum* species, could benefit from the cellulolytic action of the aforementioned bacterial families because these members of the Erysipelotrichaceae have been described to metabolize products of cellulolysis, such as cellobiose and glucose, to the main end products lactate and butyrate [68]. *Allobaculum stercoricanis* is the single known species of this genus [68]; however, *Allobaculum*-like bacteria appear to be integrated with host physiology, at least in rodents. For instance, *Allobaculum* spp. abundance was reduced in the microbiota of diet-induced obese mice [69] and positively correlated with reduced anxiety-like behavior in male mice, concomitantly with *Ruminococcus* species [70].

The Clostridiales vadinBB60 group of the Firmicutes and *Candidatus* Saccharimonas of the phylum Saccharibacteria also showed high numbers of assigned sequences. While no deeper classification of the Clostridiales group is available at present, these bacteria have been associated with the intestinal environment in mammals, for example in the mouse gut [71]. The high abundance of the enigmatic Saccharibacteria (formerly phylum TM7) in the vole microbiome is remarkable. The proportion of this taxon has been shown to increase in mice and pigs during aging [72, 73]; however, in our study no significant correlation with age was found, most likely due to the similar ages of the voles (66 to 99 days). Further studies are necessary to elucidate the functional contribution of the Saccharibacteria to the vole microbiota and whether any correlation with the aging process exists.

As the per average third-most dominant group at family level, the Bacteroidales S24-7 are further evidence of substantial overlap of the vole fecal microbiota with known microbiotas from other mammals (e.g. mouse, rabbit) and even humans [66, 74, 75]. The fermentative or nanaerobic S24-7 family appears to be almost exclusively associated with the gastrointestinal tracts of homeothermic animals where it often is among the predominant members of the microbial community [74]. Trophic guilds within this family were suggested to be specialized carbohydrate degraders that harbor enzymes involved in degradation of α-glucan, host glycan, or plant glycan. It remains to be determined whether the guild targeting plant glycans is more predominant in voles. Interestingly, the S24-7 family exhibits a very strong representation in the fecal microbiota in rex rabbits, while it is less predominant in wild mice [66, 75]. It has been suggested that, in terms of the digestive system, voles more closely resemble Lagomorphs than they do most typical rodents. It should be noted that gut microbial composition may be affected by diet, and our voles were maintained on rabbit chow, which may account for some similarities between voles and rabbits [32].

Another group of interest detected in the prairie vole microbiotas was the Melainabacteria—Gastranaerophilales, bacteria in the phylum Cyanobacteria. Melainabacteria are non-photosynthetic, obligate anaerobic fermenters that produce H_2_ and thus are thought to require syntrophic associations with H_2_ consumers, such as archeal methanogens or bacterial acetogens [50]. Vegetarian diets in humans and herbivory in other mammals seem to promote this group of bacteria; whereby fecal samples from foregut fermenting herbivores appear to harbor Melainabacteria in higher abundances than those of hindgut fermenters [50].

The large differences in the genus *Lactobacillus* abundances between animals are striking and we are currently investigating whether these are temporary fluctuations or based on microbial community characteristics. Similarly, whether the significant disparities in the abundance of Prevotellaceae Ga6A1 group, Tyzzerella 3, and Ruminiclostridium 6 are temporary and/or interrelated with the *Lactobacillus* variations remains to be determined.

Our study was conducted using animals from a captive breeding colony, and each animal was exposed only to its same-sex sibling after weaning at 20 days of age (i.e., for 46 to 79 days prior to sample collection). Under these housing conditions, microbial transmission between individuals after weaning was limited to that between cage-mates. The fact that voles require co-housing with other individuals (sibling or unrelated) complicates our assessment of microbial diversity and the role of host relatedness in microbiota composition. Future studies will need to address the effect of co-housing versus host genotype.

We opted to examine the fecal microbiota as this approach is the most useful for performing long-term studies with repeated sampling from the same animals. Studies in other species have shown that α-diversity of the microbiota was positively correlated with host density [76]. Prairie voles are known to exhibit marked population cyclicity [77], and it is unknown at what point in the population density cycle our animals were captured. Thus, the observed diversity in our study may represent a minimum or maximum estimate of diversity, relative to that found among wild populations.

The reproductive strategy of prairie voles makes them an ideal candidate for studies of parental contributions to offspring microbiota. As with many small mammal species, prairie voles display inbreeding suppression. Thus, reproduction typically occurs only between pairs of unrelated animals [12]. Since this species also displays social if not sexual monogamy, including sharing a nest [12], it would be reasonable to expect that offspring would harbor a microbiota that is a combination of that from both parents, rather than a microbiota that is driven solely by that of the mother. It would be of considerable interest to ascertain how much such a system parallels mixing of genetic material from each parent.

In several cases, also dependent on the hypervariable region studied, the relative abundances of components of the microbiotas of cage mates diverged to the point that they placed on completely different arms of a “cladogram”. Exactly why this occurred is not clear, however, it may in part be explained by patterns of coprophagy by voles. Many species consume fecal pellets. This behavior probably is most well-known in rabbits; however, it is displayed by other small rodent species as well. Coprophagy presumably serves to recover nutrients produced, but not absorbed, as the fecal material passes through the digestive tract. However, fecal pellets derived from cecal contents appear to be distinct from the more typical fecal pellets that pass directly through the gastrointestinal tract. It is possible that apparent microbial divergence between siblings in this study simply reflects inadvertent collection of cecal pellets from those two animals. The fact that two pairs of animals diverged from each other (i.e., cecal pellets from one of the animals and typical pellets from the other), while the remaining pairs displayed very similar microbiota compositions (typical fecal pellets from both animals in a pair) is about what would be expected based on the number of cecal pellets produced each day by voles [78]. Since voles display ultradian rhythms in locomotion [79], and not coincidently in cecal pellet ingestion [78], our findings suggest that pellet collection must be temporally matched between animals in future studies.

## Conclusions

Our study is, to our knowledge, the first characterization of the prairie vole fecal microbiota using a next-generation sequencing approach. As non-inbred rodents with highly developed social behavior, prairie voles provide unique challenges to microbiome analyses. Nevertheless, we were able to identify the dominant bacterial phylotypes in the vole fecal microbiota which in aggregate were remarkably consistent with the plant-derived diet of the animals. Comparative analysis also revealed the strong influences of cohabitation on the microbiota in these highly social animals. Conversely, the advantages of prairie voles as animal models in which to study social behavior could be extended towards analyses of the integration of the microbiota in the gut-brain-(social) behavior axis. Our study provides an important basis for further investigations of the interaction of the gut microbiota with the social brain.

## Supporting information

S1 Table Data analysis workflow. Worksheet with information on the data analysis pipeline.
(XLSX)

S2 Table Reference alignment to rRNA gene position conversion. Tab-delimited text file with SILVA alignment position to *E*. *coli* 16S rRNA position and hypervariable region translation.
(TXT)

S3 Table Read assignments by hypervariable region before/after removal of sequence chimeras and combining of forward and reverse reads. Binning parameters for forward/reverse read assignments to hypervariable regions are shown as E. coli 16S rRNA coordinates (for details see Material & methods).
(XLSX)

S4 Table Read Assignments by sample and hypervariable region before and after removal of sequence chimeras (usearch61). Samples and hypervariable regions are color coded as in other figures and tables in the text. The bar graph insert depicts forward and reverse read counts by hypervariable region before (blue bars) and after (red bars) removal of sequence chimeras. Reads were aligned, divided into forward and reverse reads, and assigned to the 16S rRNA hypervariable regions V2, V3, V4, V6-7, V8, and V9 as described in Materials & Methods.
(XLSX)

S5 Table Reads with OTU Assignment (Open Reference OTU Picking). The left panel shows read counts by sample and hypervariable region (HVR) following OTUs assignment using open reference OTU picking in QIIME (method usearch61, RDP classifer, SILVA 16S rRNA reference version 128, minimum count fraction 0.005%). A total of 837,386 reads received a taxonomy assignment with these parameters. Hypervariable region read count minima are highlighted in red font with light red background. The respective V2, V3, V4, V6-7, V8, and V9 sample read minima were used as rarefaction e-values during core diversity analyses. The right panel shows the reduction of read counts after quality trimming and OTU picking.
(XLSX)

S6 Table File with OTU tables. The worksheets contain a sample metadata table as well as minimum count and even sampling OTU tables from hypervariable regions V2, V3, V4, V6-7, V8, and V9. Additionally, a table with summed taxonomic assignments derived from the even sampling OTU tables is included.
(XLSX)

S1 Fig Forward and reverse read assignment frequencies to 16S rRNA gene hypervariable regions. The aligned read frequencies after binning of forward (Fig A) and reverse (Fig B) sequencing reads to hypervariable regions V2, V3, V4, V6-7, V8, and V9 are graphed (read frequencies >1) according to their relative *E*. *coli* 16S rRNA gene (SILVA accession number AB035921) positions.
(PDF)

S2 Fig Individual sample and average read distribution by 16S rRNA gene hypervariable region. The stacked bars are color-coded according to hypervariable region.
(PDF)

S3 Fig Alpha-diversity analyses by hypervariable regions. The panels depict the rarefaction curves for each sample and hypervariable region resulting from phylogenetic (PD_whole_tree) diversity, Shannon entropy (Shannon), and observed OTUs analyses with even sampling. Curves are colored by sample ID (Animal ID).
(PDF)

S4 Fig UniFrac UPGMA clustering cladograms. Unweighted (Fig A) and weighted (Fig B) UniFrac UPGMA clustering cladograms for all samples and hypervariable regions are depicted. Jackknife support values (sv) are indicated by edge colors. Sibling pairs are indicated by node color and filial generations by node shape (see legend).
(PDF)

S5 Fig UniFrac distance comparison plots. Unweighted (Fig A) and weighted (Fig B) UniFrac distance comparison plots for sibling pairs across all sequenced hypervariable regions are depicted. Non-parametric t-test p values after 1000 Monte Carlo permutations and Bonferroni correction are indicated.
(PDF)
