# Supplementary material for: Fecal microbiota in the female prairie vole (Microtus ochrogaster)
Source: PLoS One. 2018 Mar 26;13(3):e0190648. doi: 10.1371/journal.pone.0190648 (PMC5868765; doi:10.1371/journal.pone.0190648)
Supplement: S1 Fig — The aligned read frequencies after binning of forward (Fig A) and reverse (Fig B) sequencing reads to hypervariable regions V2, V3, V4, V6-7, V8, and V9 are graphed (read frequencies >1) according to their relative E. coli 16S rRNA gene (SILVA accession number AB035921) positions. (PDF) [file pone.0190648.s007.pdf]

A)

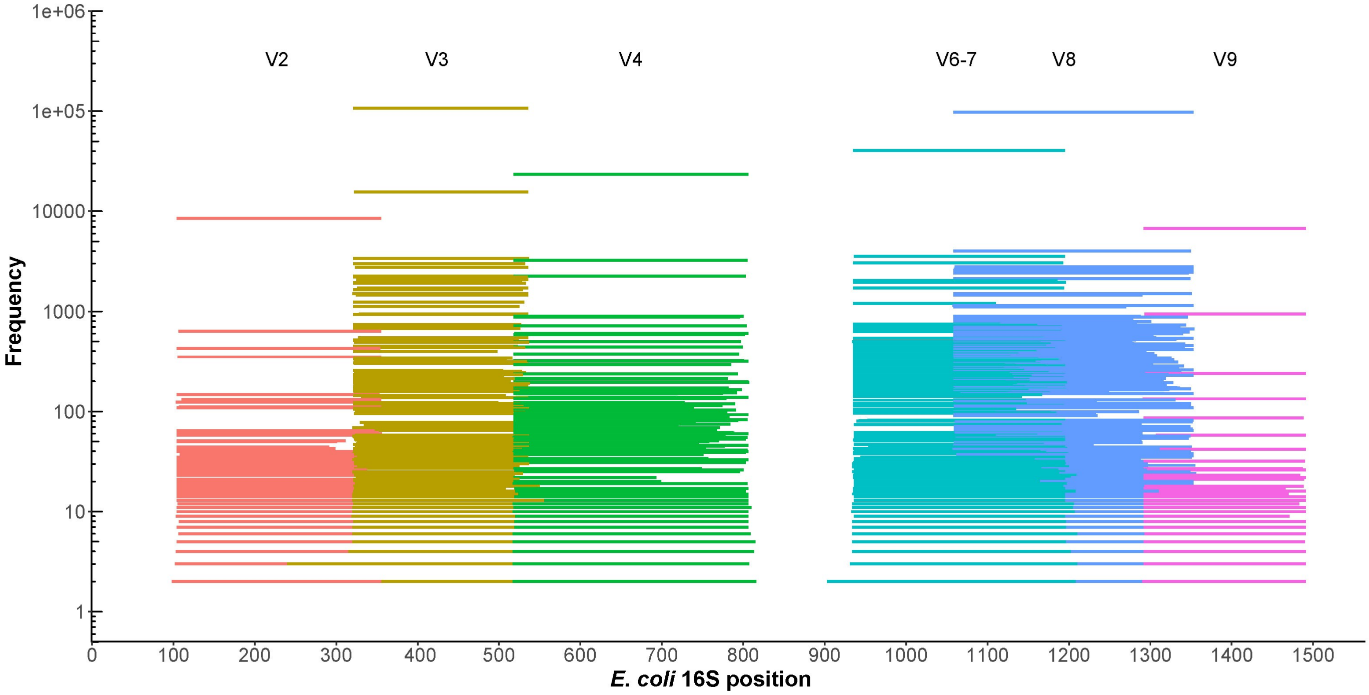

B)

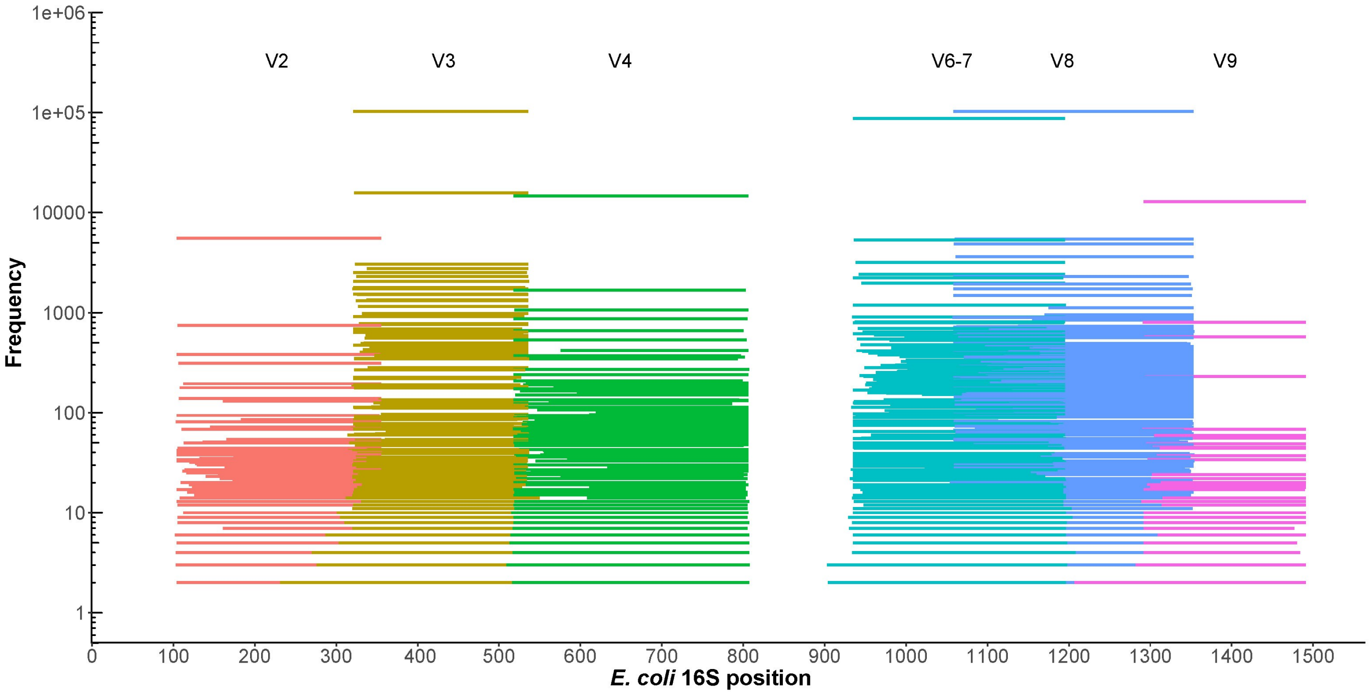

S1 Fig. Forward and reverse read assignment frequencies to 16S rRNA gene hypervariable regions. The aligned read frequencies after binning of forward (A) and reverse (B) sequencing reads to hypervariable regions V2, V3, V4, V6-7, V8, and V9 are graphed (read frequencies >1) according to their relative *E. coli* 16S rRNA gene (SILVA accession number AB035921) positions.
