# Supplementary material for: Fecal microbiota in the female prairie vole (Microtus ochrogaster)
Source: PLoS One. 2018 Mar 26;13(3):e0190648. doi: 10.1371/journal.pone.0190648 (PMC5868765; doi:10.1371/journal.pone.0190648)
Supplement: S2 Fig — The stacked bars are color-coded according to hypervariable region. (PDF) [file pone.0190648.s008.pdf]

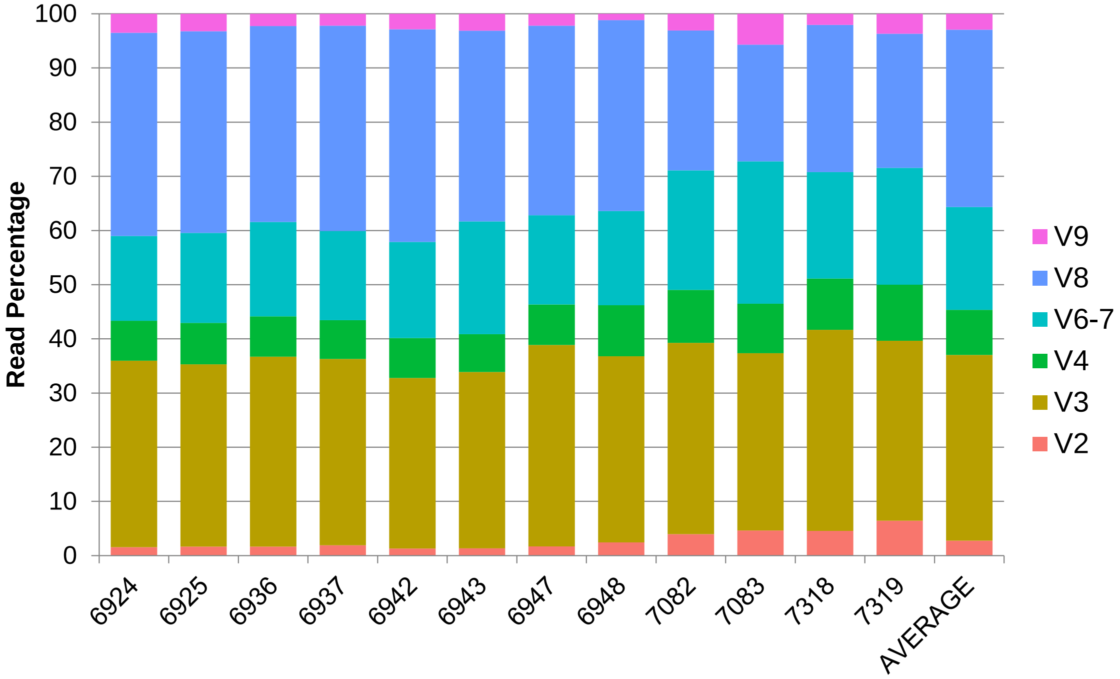

S2 Fig. Individual sample and average read distribution by 16S rRNA gene hypervariable region. The stacked bars are color-coded according to hypervariable region.
