# Supplementary material for: Fecal microbiota in the female prairie vole (Microtus ochrogaster)
Source: PLoS One. 2018 Mar 26;13(3):e0190648. doi: 10.1371/journal.pone.0190648 (PMC5868765; doi:10.1371/journal.pone.0190648)
Supplement: S3 Fig — The panels depict the rarefaction curves for each sample and hypervariable region resulting from phylogenetic (PD_whole_tree) diversity, Shannon entropy (Shannon), and observed OTUs analyses with even sampling. Curves are colored by sample ID (Animal ID). (PDF) [file pone.0190648.s009.pdf]

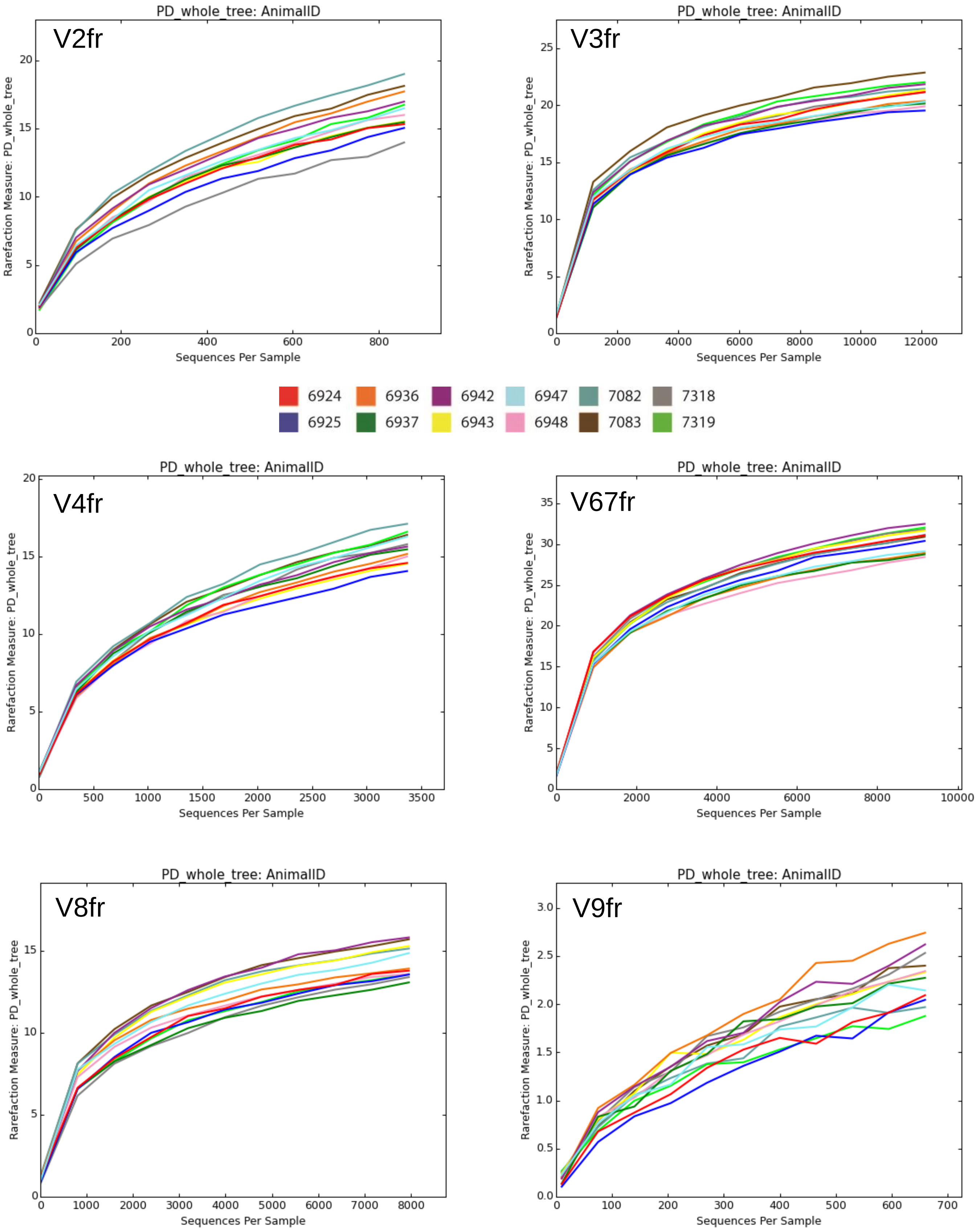
PD_whole_tree: AnimalID
V2fr
Rarefaction Measure: PD_whole_tree
Sequences Per Sample
PD_whole_tree: AnimalID
V3fr
Rarefaction Measure: PD_whole_tree
Sequences Per Sample
6924
6936
6942
6947
7082
7318
6925
6937
6943
6948
7083
7319
PD_whole_tree: AnimalID
V4fr
Rarefaction Measure: PD_whole_tree
Sequences Per Sample
PD_whole_tree: AnimalID
V67fr
Rarefaction Measure: PD_whole_tree
Sequences Per Sample
PD_whole_tree: AnimalID
V8fr
Rarefaction Measure: PD_whole_tree
Sequences Per Sample
PD_whole_tree: AnimalID
V9fr
Rarefaction Measure: PD_whole_tree
Sequences Per Sample

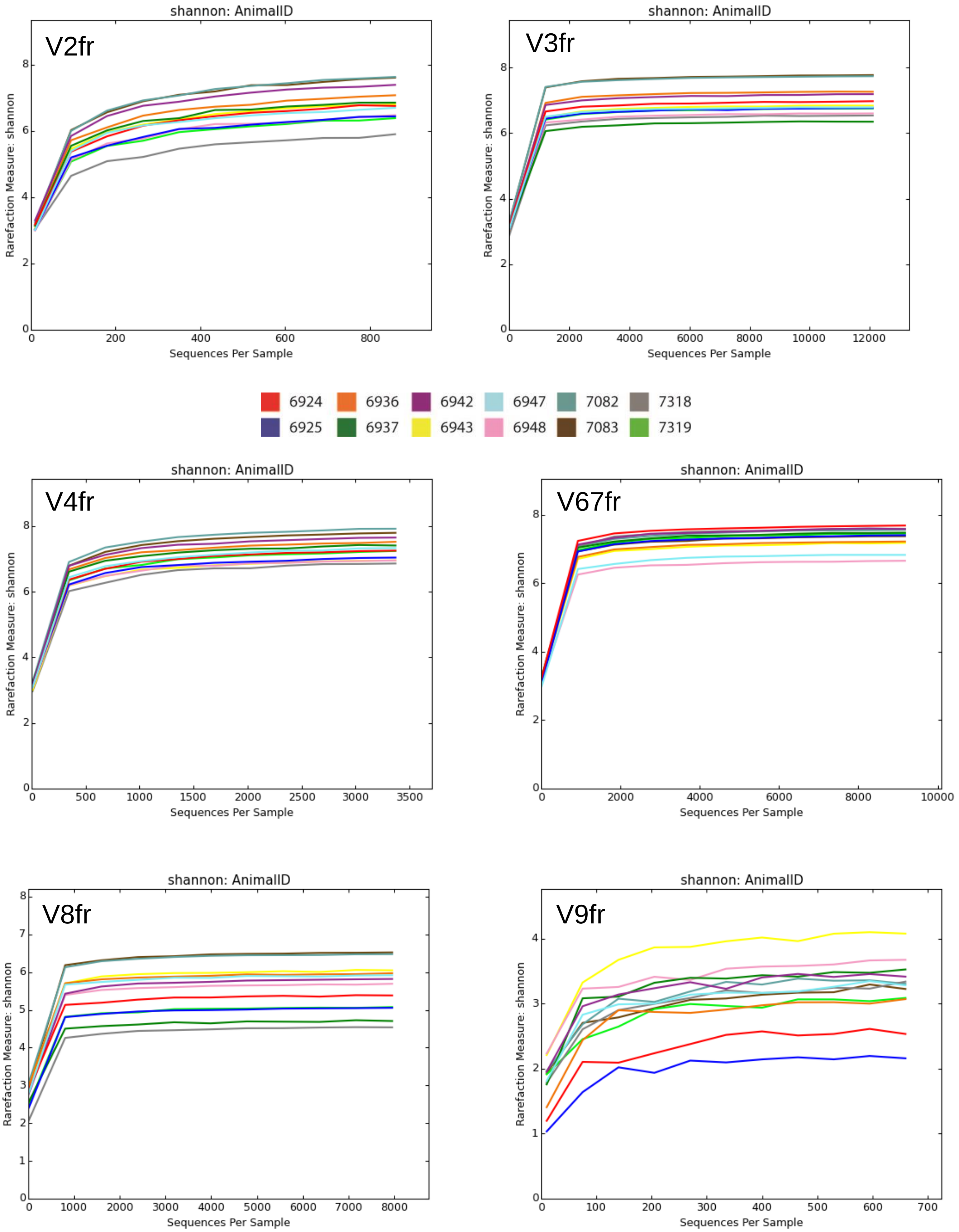
shannon: AnimalID
V2fr
V3fr
V4fr
V67fr
V8fr
V9fr
Rarefaction Measure: shannon
Sequences Per Sample
6924
6925
6936
6937
6942
6943
6947
6948
7082
7083
7318
7319

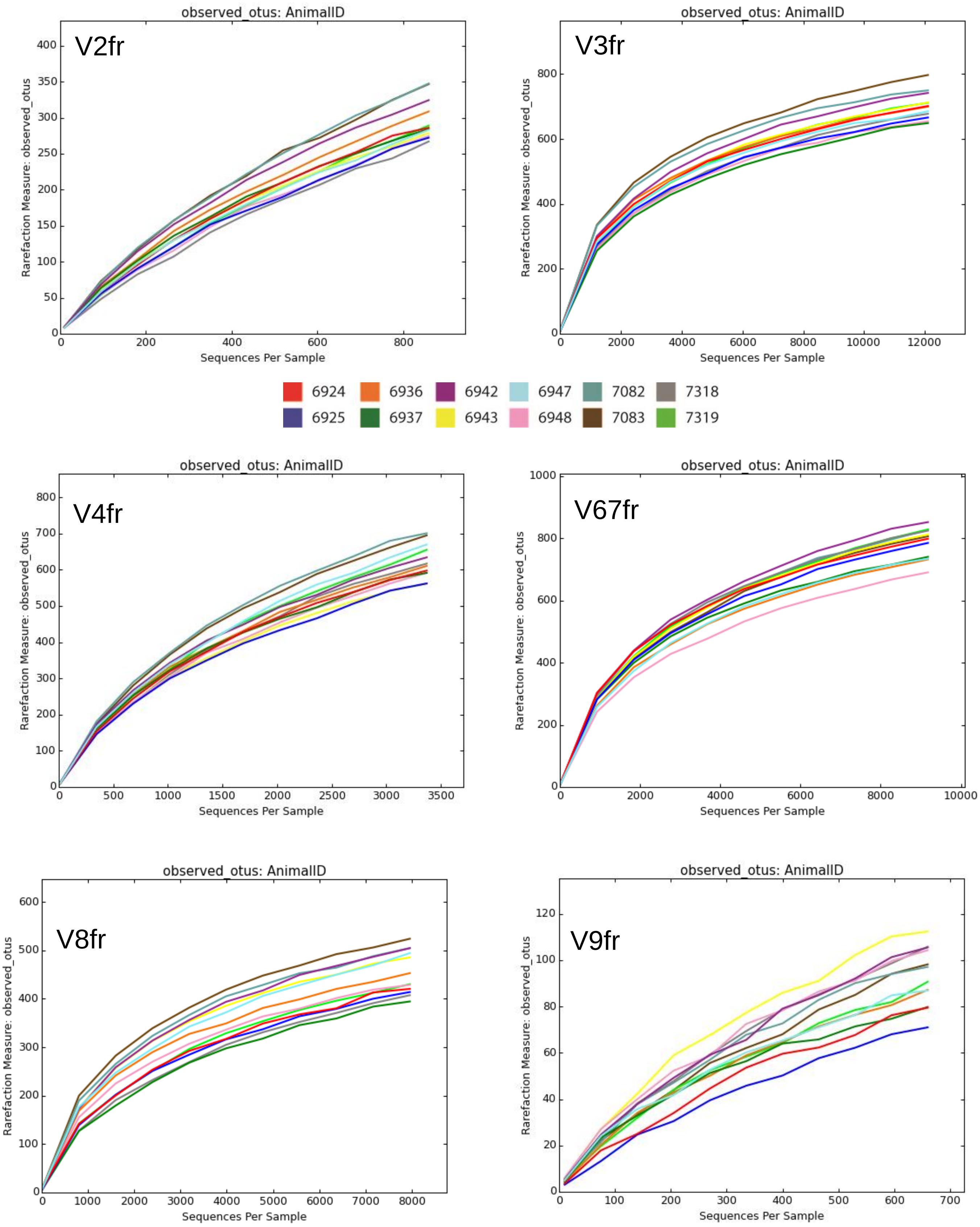

S3 Fig. Alpha-diversity analyses by hypervariable regions. The panels depict the rarefaction curves for each sample and hypervariable region resulting from phylogenetic (PD_whole_tree) diversity, Shannon entropy (Shannon), and observed OTUs analyses with even sampling. Curves are colored by sample ID (Animal ID).
