# Supplementary material for: Fecal microbiota in the female prairie vole (Microtus ochrogaster)
Source: PLoS One. 2018 Mar 26;13(3):e0190648. doi: 10.1371/journal.pone.0190648 (PMC5868765; doi:10.1371/journal.pone.0190648)
Supplement: S4 Fig — Unweighted (Fig A) and weighted (Fig B) UniFrac UPGMA clustering cladograms for all samples and hypervariable regions are depicted. Jackknife support values (sv) are indicated by edge colors. Sibling pairs are indicated by node color and filial generations by node shape (see legend). (PDF) [file pone.0190648.s010.pdf]

A)

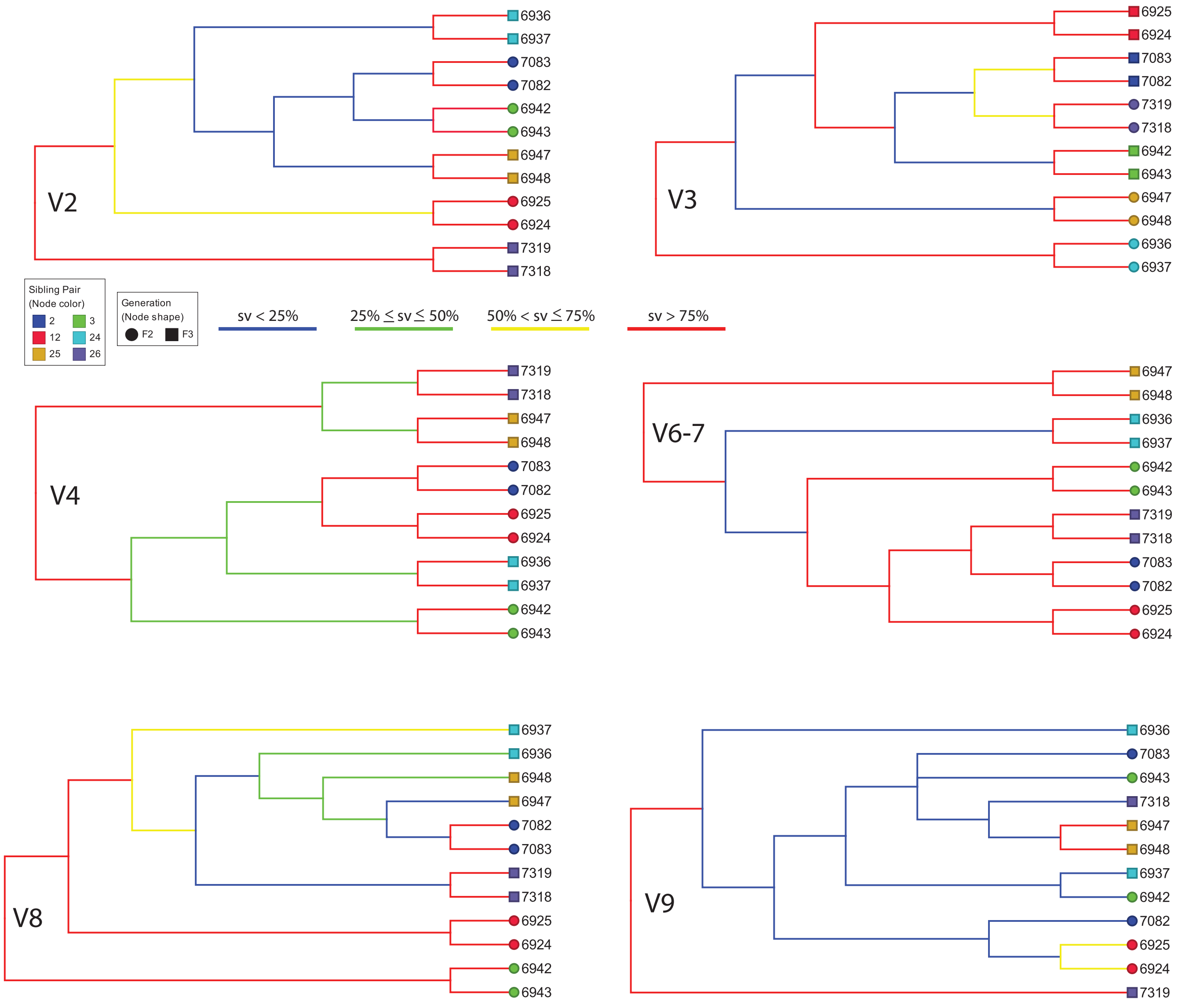

V2
V3
V4
V6-7
V8
V9
Sibling Pair
(Node color)
2
3
12
24
25
26
Generation
(Node shape)
F2
F3
sv < 25%
25% ≤ sv ≤ 50%
50% < sv ≤ 75%
sv > 75%
6936
6937
7083
7082
6942
6943
6947
6948
6925
6924
7319
7318

B)

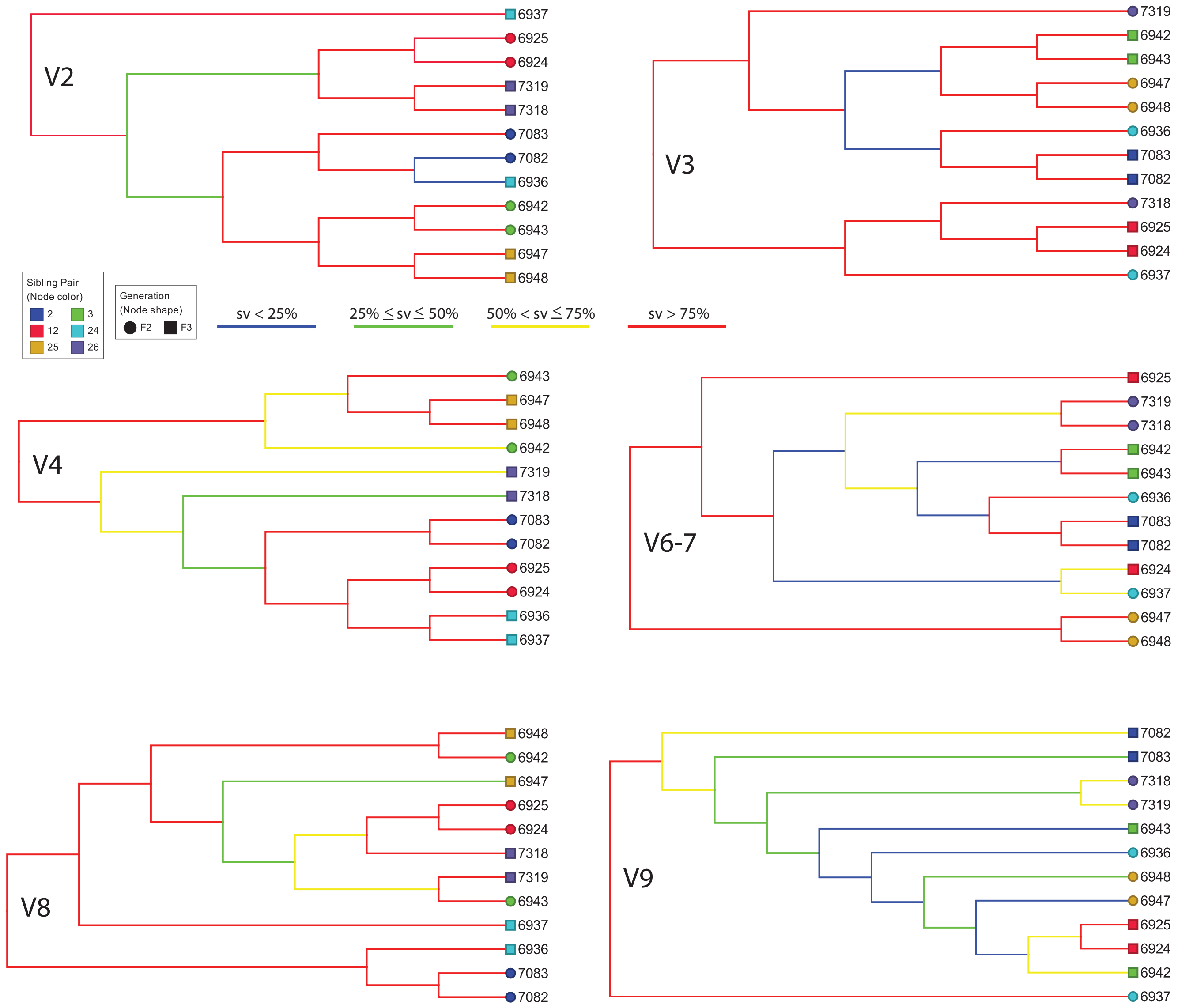

S4 Fig. UniFrac UPGMA clustering cladograms.
Unweighted (A) and weighted (B) UniFrac UPGMA clustering cladograms for all samples and hypervariable regions are depicted. Jackknife support values (sv) are indicated by edge colors. Sibling pairs are indicated by node color and filial generations by node shape (see legend).
