# Supplementary material for: Fecal microbiota in the female prairie vole (Microtus ochrogaster)
Source: PLoS One. 2018 Mar 26;13(3):e0190648. doi: 10.1371/journal.pone.0190648 (PMC5868765; doi:10.1371/journal.pone.0190648)
Supplement: S5 Fig — Unweighted (Fig A) and weighted (Fig B) UniFrac distance comparison plots for sibling pairs across all sequenced hypervariable regions are depicted. Non-parametric t-test p values after 1000 Monte Carlo permutations and Bonferroni correction are indicated. (PDF) [file pone.0190648.s011.pdf]

A)

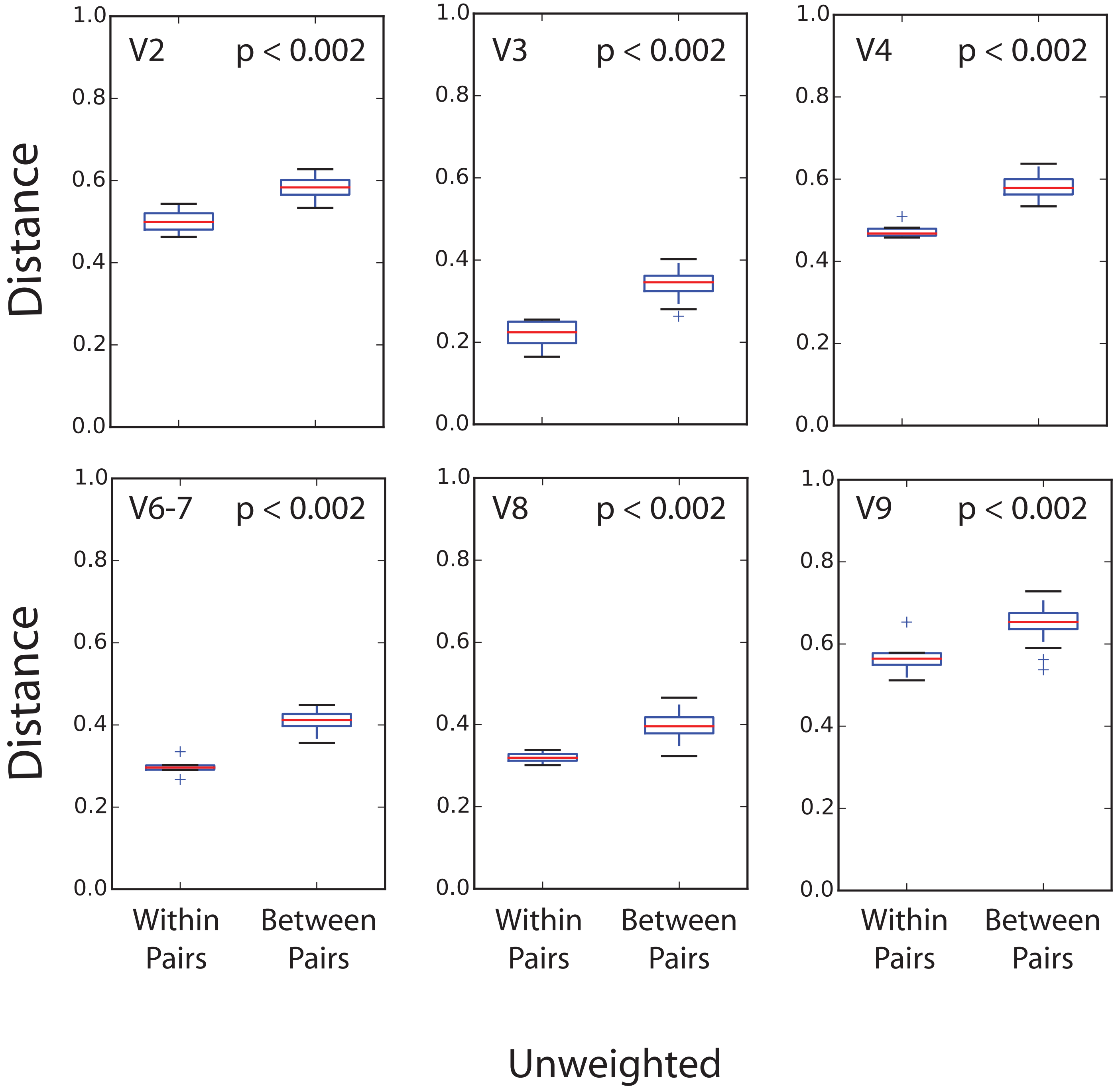

V2
p < 0.002
V3
p < 0.002
V4
p < 0.002
V6-7
p < 0.002
V8
p < 0.002
V9
p < 0.002
Distance
Distance
1.0
0.8
0.6
0.4
0.2
0.0
Within Pairs
Between Pairs
Unweighted

B)

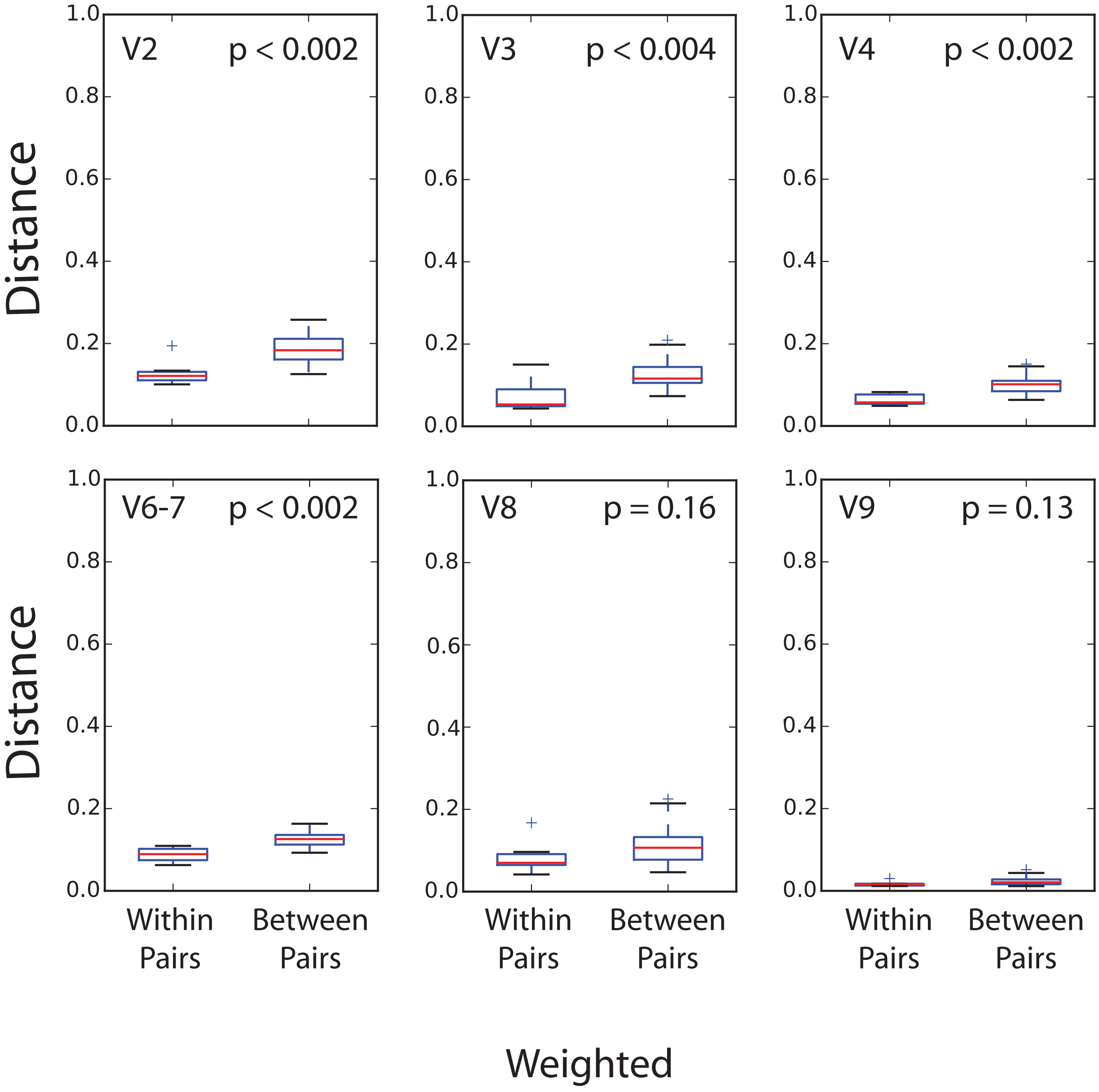

S5 Fig. UniFrac distance comparison plots.

Unweighted (A) and weighted (B) UniFrac distance comparison plots for sibling pairs across all sequenced hypervariable regions are depicted. Non-parametric t-test p values after 1000 Monte Carlo permutations and Bonferroni correction are indicated.
